# Nitrogen oxide cycle regulates nitric oxide levels and bacterial cell signaling

**DOI:** 10.1038/srep22038

**Published:** 2016-02-25

**Authors:** Yasuyuki Sasaki, Haruka Oguchi, Takuya Kobayashi, Shinichiro Kusama, Ryo Sugiura, Kenta Moriya, Takuya Hirata, Yuriya Yukioka, Naoki Takaya, Shunsuke Yajima, Shinsaku Ito, Kiyoshi Okada, Kanju Ohsawa, Haruo Ikeda, Hideaki Takano, Kenji Ueda, Hirofumi Shoun

**Affiliations:** 1 Department of Bioscience, Faculty of Applied Bioscience, Tokyo University of Agriculture, Sakuragaoka Setagaya-ku, Tokyo 156-8502, Japan; 2 Faculty of Life and Environmental Sciences, University of Tsukuba, Tsukuba, Ibaraki, Japan; 3 Laboratory of Microbial Engineering, Kitasato Institute for Life Sciences, Kitasato University, 1-15-1 Kitasato, Sagamihara, Kanagawa 228-855, Japan; 4 Life Science Research Center, College of Bioresource Sciences, Nihon University, 1866 Kameino, Fujisawa 252-0880, Japan

## Abstract

Nitric oxide (NO) signaling controls various metabolic pathways in bacteria and higher eukaryotes. Cellular enzymes synthesize and detoxify NO; however, a mechanism that controls its cellular homeostasis has not been identified. Here, we found a nitrogen oxide cycle involving nitrate reductase (Nar) and the NO dioxygenase flavohemoglobin (Fhb), that facilitate inter-conversion of nitrate, nitrite, and NO in the actinobacterium *Streptomyces coelicolor*. This cycle regulates cellular NO levels, bacterial antibiotic production, and morphological differentiation. NO down-regulates Nar and up-regulates Fhb gene expression via the NO-dependent transcriptional factors DevSR and NsrR, respectively, which are involved in the auto-regulation mechanism of intracellular NO levels. Nitrite generated by the NO cycles induces gene expression in neighboring cells, indicating an additional role of the cycle as a producer of a transmittable inter-cellular communication molecule.

Nitric oxide (NO) is a freely diffusible neutral gas that acts as an important signaling molecule to control metabolic pathways in bacteria and higher eukaryotes. Since NO is highly reactive and toxic for living cells, cells must have strict control over intracellular NO levels. The genus *Streptomyces* includes bacteria that produce many commercially useful secondary metabolites that are extremely important to humans. They follow an elaborate life cycle that includes vegetative (or substrate) mycelial growth, aerial mycelial growth, and sporulation. Recent studies suggest that actinobacteria require NO to regulate various metabolic pathways^1^,^2^,^3^,^4^,^5^, however, little is known about the mechanism by which actinobacteria generate NO, except for NO synthase (NOS) distribution in a limited number of actinobacterial species.

Recently, we reported a unique nitrogen metabolism in *Streptomyces antibioticus*, in which organic nitrogen was mineralized to form nitrogen oxide species, nitrite (NO_2_^−^), nitrate (NO_3_^−^), and NO during vegetative cell growth under aerobic conditions, and NO_2_^−^ was excreted into the medium^6^. Since arginine analogs inhibited the production of NO_2_^−^ and NO, we suggested that NOS is involved in the NO_2_^−^ -formation, and proposed a NO_2_^−^ -forming pathway (pathway 1, below), although presence of a NOS enzyme has not been demonstrated in *S. antibioticus*.


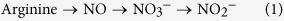


We also showed that *S. antibioticus* produces a large amount of flavohemoglobin (Fhb) and oxidizes NO to generate nitrate (NO_3_^−^)^7^. The bacterium produces Fhb without exogenous nitrosative stress under NO_2_^−^-producing conditions, which is in contrast to most bacteria that produce Fhb only in the presence of exogenous NO^8^. Membrane-bound NO_3_^−^ reductase (Nar) catalyzes the last step in the NO_2_^−^-forming pathway. Some bacteria produce Nar in the presence of NO_3_^−^ for anaerobic respiration (denitrification)^9^, while *S. antibioticus* produces Nar and NO_2_^−^ in the absence of exogenous NO_3_^−^ under aerobic conditions^6^,^10^. Thus, both Fhb and Nar are produced in *S. antibioticus* to form NO_2_^−^ under conditions that are different from those known in many other bacteria. Given that production of NO_2_^−^ synchronized with rapid cell growth and was inhibited by glucose or glycerol, we suggested that the “NO_2_^−^-forming pathway” is an energy-producing metabolic reaction, although this conclusion requires further investigation.

This study investigated NO_2_^−^ production by a model actinobacterium, *S. coelicolor* A3(2). Although *S. coelicolor* A3(2) produces and excretes NO_2_^−^ like *S. antibioticus*, the NO-producing mechanism is likely to be different from the latter since the *S. coelicolor* genome^11^ does not encode a gene for NOS. In addition, no gene encoding the dissimilatory, NO-generating nitrite reductase was found in the genome. Here we demonstrated a NO-formation via NO_2_^−^ produced from organic nitrogen, and homeostatic regulation of cellular NO in *S. coelicolor*. The endogenously formed NO is controlled by the nitrogen oxide cycle and acts as a signaling molecule for antibiotic production and morphological differentiation.

## Results

### *S. coelicolor* nitrogen-oxide cycle tunes endogenous NO concentration

**Figure 1:**
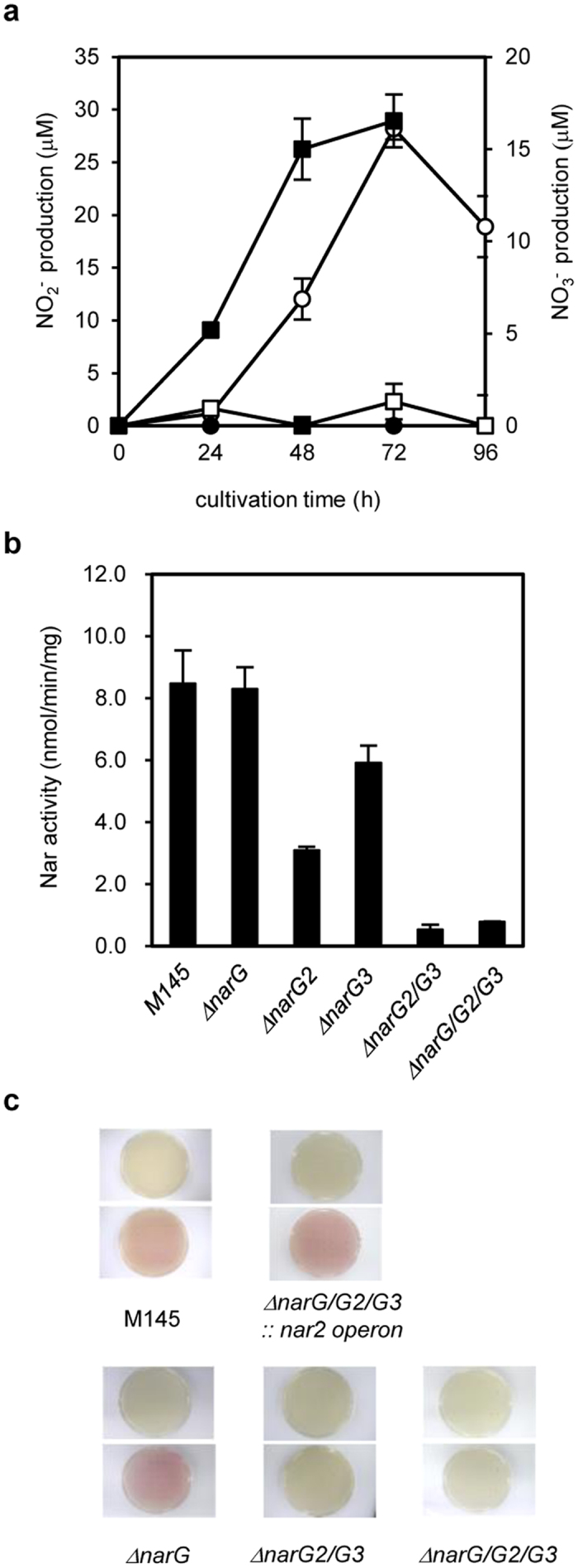
Nitrite and nitrate production by *S. coelicolor A3(2)* M145 and its derivatives. (**a**) Spores of *S. coelicolor A3(2)* M145 or its defective strains were inoculated on cellophane-covered YEME-gln plates at intervals of 1 cm (a total of 45 spots). Levels of NO_2_^−^ and NO_3_^−^ extracted from the medium plate at each cultivation time as described in Materials and Methods were determined. Open circle, NO_2_^−^ production by M145; closed circle, NO_3_^−^ by M145; open square, NO_2_^−^ by ∆*narG/G2/G3*; closed square, NO_3_^−^ by ∆*narG/G2/G3*. (**b**) Nar activity in the membrane fraction of M145 and its derivatives. Cells were cultivated as described in **(a)** for 48 h and the membrane fraction was prepared as described in METHODS. (**c**) Each strain was cultivated as in (**a**) except that spores were inoculated directly on the agar plate (without cellophane). Photographs of each strain: upper line, grown cells without treatment; lower line, NO_2_^−^ production was visualized by dyeing with 1 ml Griess reagent. Error bars indicate standard deviation (n = 3).

We found that *S. coelicolor* A3 (2) M145 (M145) excreted NO_2_^−^ into the medium when cultured with organic nitrogen as the nitrogen source under NO_2_^−^-producing conditions^6^ (Fig. 1a). The NO_2_^− _^production was also observed when M145 was grown on minimal medium containing L-asparagine as the sole nitrogen source (chemically defined medium) (supplementary Fig. 1), showing that M145 can convert organic nitrogen to NO_2_^−^. NO_2_^−^ concentration increased for 72 h, indicating that *S. coelicolor* produces NO_2_^−^ from organic nitrogen during the early vegetative growth phase. The *S. coelicolor* A3(2) has three Nar enzyme homologs (Nar1, Nar2, and Nar3 encoded by *narGHJI*, *narG2H2J2I2*, and *narG3H3J3I3*, respectively). We constructed mutants lacking combinations of the three *narG* paralogs, each of which encodes the catalytic subunits of Nar. (∆*narG*, ∆*narG2*, ∆*narG3*, ∆*narG2/G3*, and ∆*narG/G2/G3*). The wild-type M145 strain cultured under the NO_2_^−^-producing conditions showed Nar activity in cell membrane fractions (Fig. 1b). The ∆*narG2/G3* and ∆*narG/G2/G3* mutants showed drastic decrease in this activity (Fig. 1b) accompanied by decreased NO_2_^−^ production (Fig. 1c). The ∆*narG/G2/G3* mutant excreted NO_3_^−^ instead of NO_2_^−^ (Fig. 1a), indicating that Nar reduces NO_3_^−^ to NO_2_^−^. Introducing the *narG2H2J2I2* operon into the ∆*narG/G2/G3* mutant restored the NO_2_^−^-production (Fig. 1c). ∆*narG* mutant produced almost the same level of Nar activity as the wild type M145 strain (Fig. 1c). The result indicated that *narG2* and *narG3* but not *narG* are responsible for NO_2_^−^ formation from organic nitrogen in the culture medium. Recently, Fischer *et al.* proposed that all three Nars are synthesized in *S. coelicolor* during the aerobic growth independent of the presence of NO_3_^−^, and they further indicated that *narG* is mainly working in spore, *narG2* and *narG3* are mainly working in mycelium, respectively. Thus, present results of *narG2* and *narG3* expression correspond with the results reported by Fischer *et al.*^12^,^13^.

**Figure 2:**
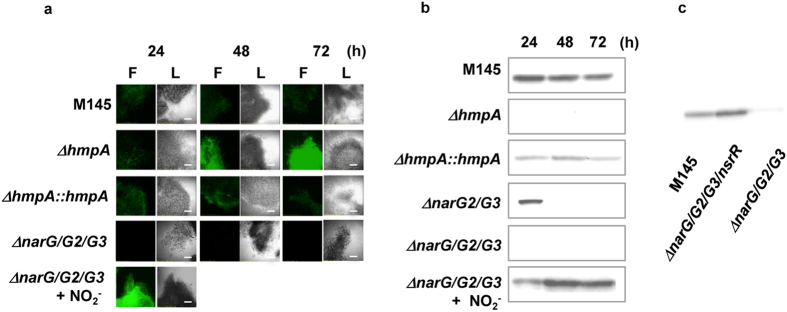
Production of endogenous NO and NO dioxygenase (Fhb) in *S. coelicolor*. (**a**) Spores of each strain were inoculated at the center of a YEME-gln medium plate (one spot, without cellophane) and cells were grown for the indicated period. Cells were then collected with a toothpick and treated with 10 μM DAF-2DA as described previously. NO_2_^−^ (50 μM) was added into the growth medium (bottom figure). hotographs under visible light (L) or fluorescence (F) were taken using FLUOVIEW FV300 System. Scale bar, 100 μm (**b**) Soluble proteins (7 μg) from each strain grown for the indicated period were used to detect Fhb production by western blotting. NO_2_^−^ (50 μM) was added into the growth medium (bottom figure). (**c**) Production of FhbA is controlled by the negative transcriptional factor NsrR. Cells were cultivated as in (**a**) for 48 h. Soluble proteins (7 μg) of each strain were prepared as in (**b**).

Since NO_2_^−^ as well as NO_3_^−^ are produced as endogenously oxidative metabolites of NO, we investigated NO accumulation by using a fluorometric method and observed that NO production activity was present in M145 (Fig. 2a). The gene knockout of *hmpA* (SCO7472, encodes FhbA) (∆*hmpA*) accumulated more NO than did the M145 strain (Fig. 2a), and complementation with the *hmpA* gene (∆*hmpA:: hmpA*) attenuated NO accumulation to the same level as that in M145 (Fig. 2a). Western blot analysis detected the *hmpA* gene product (flavohemoglobin, Fhb) in the NO_2_^−^-producing cells (Fig. 2b), indicating that the NO dioxygenase activity of Fhb^8^ led to lowered intracellular NO levels.

When grown under the NO_2_^−^-producing conditions, the ∆*narG/G2/G3* cells accumulated little NO, whereas exogenous addition of NO_2_^−^ to the cells restored the NO formation (Fig. 2a). This showed that NO was formed from NO_2_^−^, and that the ∆*narG/G2/G3* cells could not form NO because they could not produce NO_2_^−^ (Fig. 1a,c). The ∆*narG/G2/G3* cells produced little Fhb, and addition of an NO donor (50 μM NO_2_^−^) to the medium restored its production (Fig. 2b). Transcription of *hmp* is known to be negatively regulated by NsrR^14^,^15^, which loses the ability to repress *hmp* upon exposure to NO. Disruption of *nsrR* in the ∆*narG/G2/G3* cells also recovered the defect of FhbA production (Fig. 2c). These results are consistent with the notion that NO is formed from NO_2_^−^ that is produced by Nar and derepresses Fhb production through NsrR transcription control. The present results demonstrate that Nar encoded by *narG2* and *narG3* reduces NO_3_^−^ to NO_2_^−^, which is converted to NO, while Fhb oxidizes NO to NO_3_^−^. These enzymes constitute the metabolic cycle of nitrogen oxides, which can participate in the cellular processes without exogenous supply of either of the nitrogen oxides.

The expression of FhbA was observed in ∆*narG2/G3* mutant only at early stage of growth (24 h) (Fig. 2b). This suggests that *narG* supported the expression of FhbA, confirming that *narG* acts during at spore germination stage and also confirms that *narG2* and *narG3* are working during vegetative growth phase to supply NO via NO_2_^−^.

### Endogenous NO promotes antibiotic production and regulates differentiation

**Figure 3:**
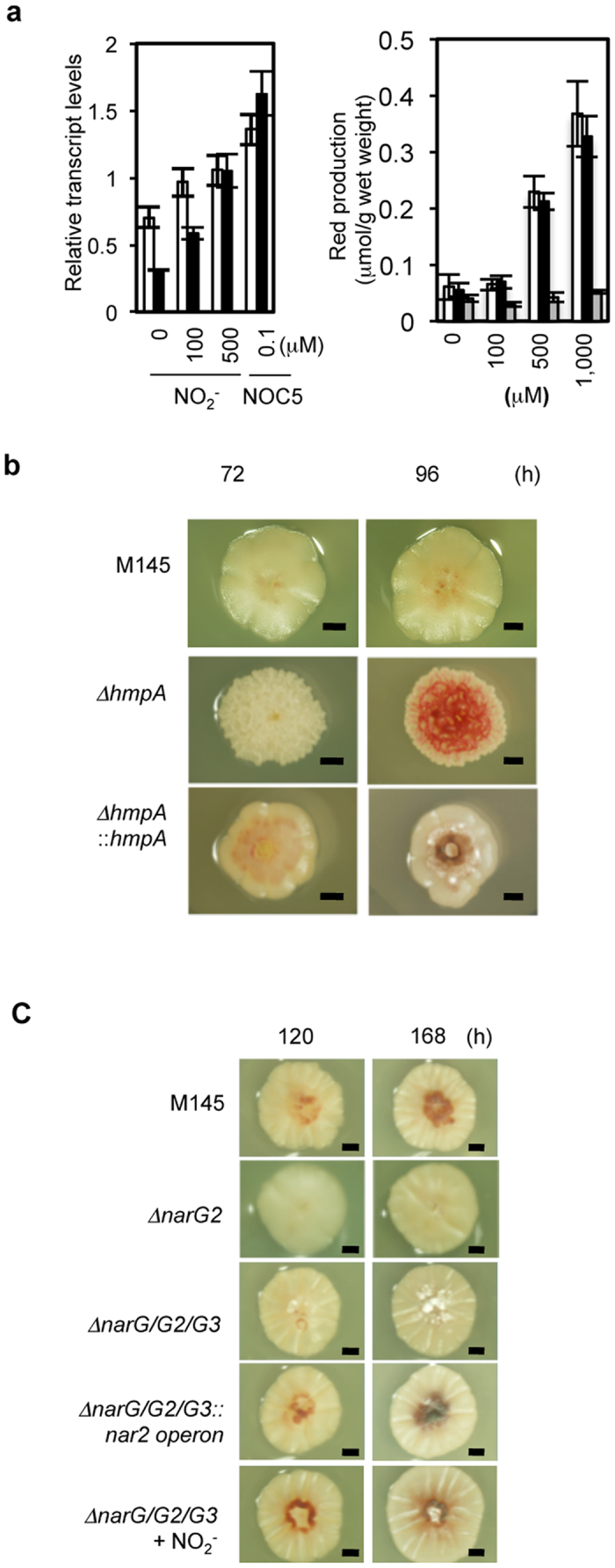
NO derived from NO_2_^−^ regulates antibiotic (RED) production and morphological differentiation in *S. coelicolor*. Cells were grown for the indicated period as in Fig. 2 except in panel (**a**). Scale bar, 1 mm. (**a**) NO_2_^−^- or NO-dependent *redD* expression (left panel) or RED production (right panel). Cells were grown for 48 h as in Fig. 1a, and cells on the cellophane membrane were transferred to a fresh medium plate and further incubated. Left panel, cells were incubated at 30 °C for 60 (NO_2_^−^) or 25 (NOC5) min, total RNA was extracted, and expression was determined by qPCR. Right panel, effects of NO_2_^−^ on RED production, calculated as μmol RED/g wet weight cells. White bar, wild M145; dark bar, ∆*narG/G2/G3*; gray bar, ∆*redD*. Error bars indicate standard deviation (n = 3). (**b**) Phenotypes of *hmpA* deletion mutant. (**c**) Phenotypes of *narG*s mutants. Bottom, complementation of RED production and abnormal differentiation in the *narG/G2/G3* mutant by exogenous NO_2_^−^ (100 μM NO_2_^−^ was added to the medium during culture). (**d**) Involvement of Nar in conversion of NO_2_^−^ to NO. Cells were grown in the presence of 500 (left column) or 1,000 μM (right column) NO_2_^−^.

Addition of NO donors to *S. coelicolor* cultures up-regulated transcription of *redD*, which encodes a positive regulator of undecylprodigiosin (Red) synthesis genes^16^ and Red production (Fig. 3a). Direct supply of NO by NOC5 induced *redD* transcription more effectively than NO_2_^−^ (Fig. 3a, left), which indicates that NO directly regulates *redD* transcription. The ∆*hmpA* strain produced more Red than did the M145 strain (Fig. 3b) whereas ∆*narG2* and ∆*narG/G2/G3* mutants produced little Red (Fig. 3c) in accordance with the roles of Fhb and Nar in sequestering and producing NO, respectively. Addtion of NO donor (100 μM NO_2_^−^) restored the Red production by the ∆*narG/G2/G3* mutant (Fig. 3c), showing that NO_2_^−^ production from NO_3_^−^ by Nar is necessary for NO formation. Higher concentration of NO_2_^−^ increased Red production by the M145 and ∆*narG/G2/G3* strains, but *redD* gene knockout confirms no NO-dependent Red production (Fig. 3a, right). These results indicated that the bacterial Nar and FhbA control cellular NO levels and regulate Red production. Moreover, the ∆*narG/G2/G3* mutant developed aerial mycelia after cultivation for 120 h, which is much earlier than differentiation of aerial mycelia in M145. Addition of 100 μM NO_2_^−^ to the medium recovered this phenotype (Fig. 3c), indicating that endogenously generated NO delays aerial mycelia development.

### DevSR controls nitrogen oxide cycle and cellular NO levels

**Figure 4:**
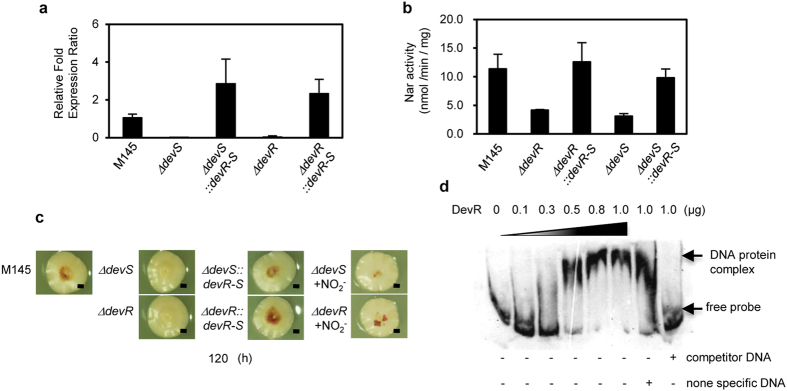
A DevSR two-component system controls *narG2* gene expression in *S. coelicolor*. Cells were grown on a cellophane membrane as in Fig. 1a for 72 h, and transcriptional analysis of *narG2* using real-time PCR (**a**) and measurement of total Nar activity in the membrane fraction (**b**) were performed. (**c**) *dev*S and *dev*R mutants exhibit the same phenotype as ∆*narG2* (Fig. 3c), and the phenotype is canceled by exogenous 500 μM NO_2_^−^. (**d**) Reaction mixtures (10 μl) containing 400 nmol DIG-labeled oligonucleotide probe and recombinant DevR (0.1, 0.3, 0.5, 0.8, and 1.0 μg) were incubated for 30 min at 37 °C and separated by electrophoresis in a native 6% polyacrylamide gel. Unlabeled probe (200 ng) was used as competitor DNA, and 200 ng of the control unlabeled oligonucleotide provided by DIG Gel Shift Kit 2^nd^ generation was used as nonspecific DNA.

DevS is a heme-containing, NO-sensing histidine kinase^17^,^18^ that transduces signals to a transcriptional regulator DevR, constituting a two-component system (TCS) with DevS. Deletion of the *S. coelicolor* orthologs *devS* (SCO203) or *devR* (SCO204) (Supplementary Fig. 2) decreased transcripts of *narG2* in *S. coelicolor* along with reducing Nar activity (Fig. 4a,b). Both the deletion mutants produced low levels of Red as observed in the ∆*narG2* mutant and were recovered by a high concentration of NO_2_^−^ (Fig. 4c). In addition, DevR (rDevR) protein could bind to the upstream region of *narG2 in vitro* (Fig. 4d). These results revealed that the *S. coelicolor* DevSR regulates the expression of *narG2* and are integral components in the NO-forming pathway.

**Figure 5:**
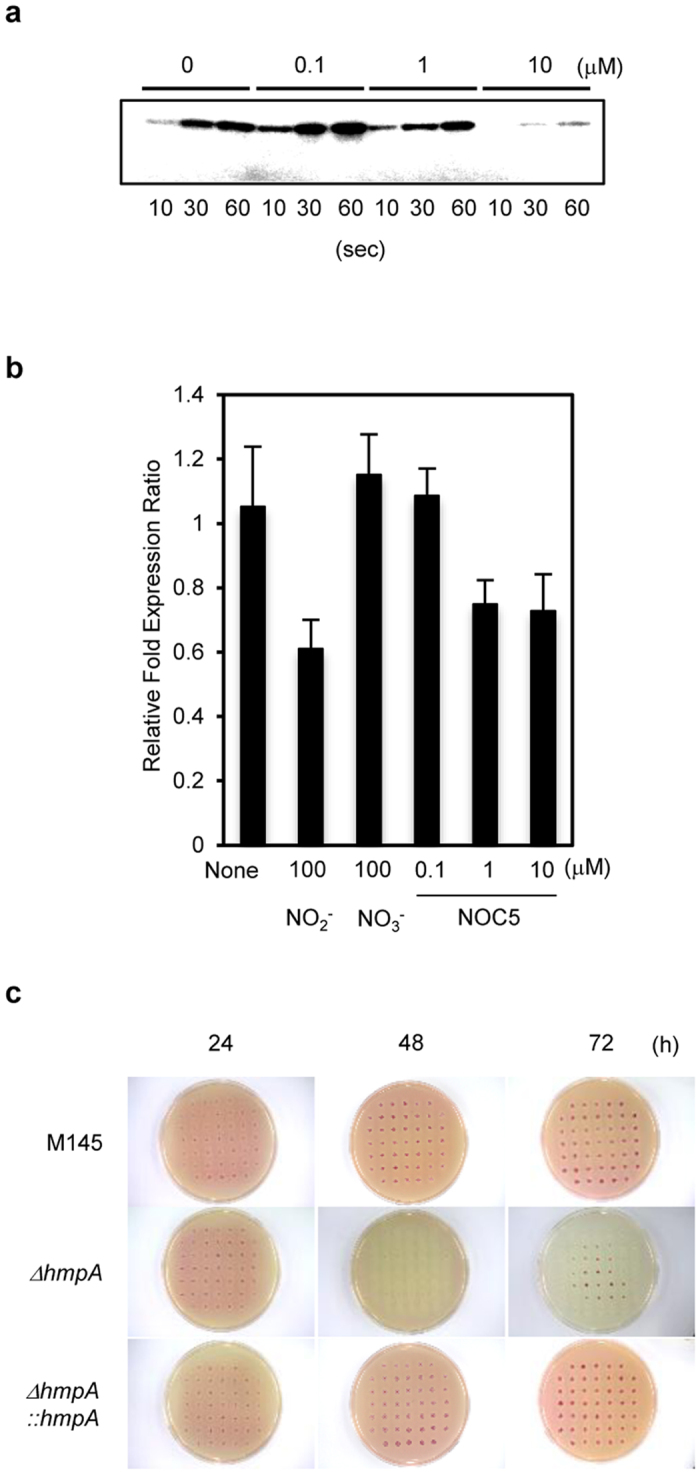
Endogenous NO controls its own intracellular concentration. (**a**) Autophosphorylation of recombinant DevS on incubation with NOC5. Purified rDevS was incubated with ^32^P-γ-ATP in the presence of 0–10 μM NOC5 for the indicated time (10–30 s). (**b**) Effects of NO on the transcription of *narG2* in the wild-type M145 strain. Cells were grown for 48 h as in Fig. 3a (left) and transferred to a fresh medium containing 100 μM NO_2_^−^ or NO_3_^−^ or the indicated concentration of NOC5 (0–10 μM); then cells were incubated for 1 h (NO_2_^−^ or NO_3_^−^) or 25 min (NOC5), and total RNA was extracted from the cells and subjected to qPCR. Error bars indicate standard deviation (n = 3). (**c**) Excess endogenous NO stops NO_2_^−^ production. Cells were grown and NO_2_^−^ was stained as in Fig. 1c.

*In vitro* phosphorylation of rDevS, induced by its autokinase activity (Fig. 5a), was decreased in the presence of more than 1 μM NOC5. Exposure to high concentrations of exogenous NO_2_^−^ or NOC5 also inhibited cellular transcripts of *narG2* (Fig. 5b) and Nar activity (Supplementary Fig. 3). These results indicate that NO negatively regulates the DevSR TCS and transcription of the *narG2* operon. The ∆*hmpA* mutant which accumulated more NO in the cell (Fig. 2a) excreted less NO_2_^−^ into the medium after cultivation for 48 h (Fig. 5c), and the defect was complemented by introduction of *hmpA* gene (Fig. 5c). This observation supports the inhibitory effect of excess intracellular NO on *narG2* expression since NO_2_^−^ is a product of Nar reaction.

It can be concluded from these results that the DevSR TCS system regulates the concentration of endogenous NO by controlling the expression of the *nar2* gene cluster and that NO itself acts as the negative regulator depending on its intracellular concentration.

### NO_2_^−^ is an intercellular signaling molecule

**Figure 6:**
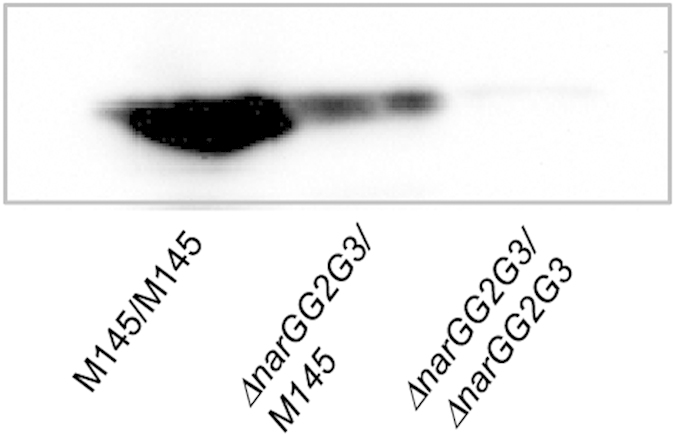
Cell-to-cell communication mediated by NO_2_^−^. Spores of M145 or *narGs* mutant were inoculated onto the YEME-gln plate (a total of 9 spots) and incubated for 48 h. Only cells growing in the center spot were collected and subjected to western blotting to detect the production of Fhb. The M145/M145, M145 strain was inoculated at all 9 spots; ∆*narGG2G3*/M145; M145 was inoculated in all spots except the center spot in which the ∆*narG/G2/G3* mutant was inoculated; the ∆*narGG2G3/*∆*narGG2G3*, ∆*narG/G2/G3* mutant was inoculated at all spots.

When the ∆*narG/G2/G3* strain was cultured on a plate in which a single colony was surrounded by eight colonies of the parent strain M145, ∆*narG/G2/G3* that had lost its Fhb-producing ability (Fig. 2) began to produce FhbA again (Fig. 6). This indicated that excreted NO_2_^−^ or NO derived from NO_2_^−^ acts as a signaling molecule for communication between cells, and confirmed that NO is the end product of the nitrogen oxide cycle and is a hormone-like molecule. We found that Red-producing ability of the ∆*narG/G2/G3* mutant strain was not restored (Supplementary Fig. 4), probably because NO_2_^−^ transmitted to the mutant could not supply sufficient NO to trigger Red synthesis.

## Discussion

**Figure 7:**
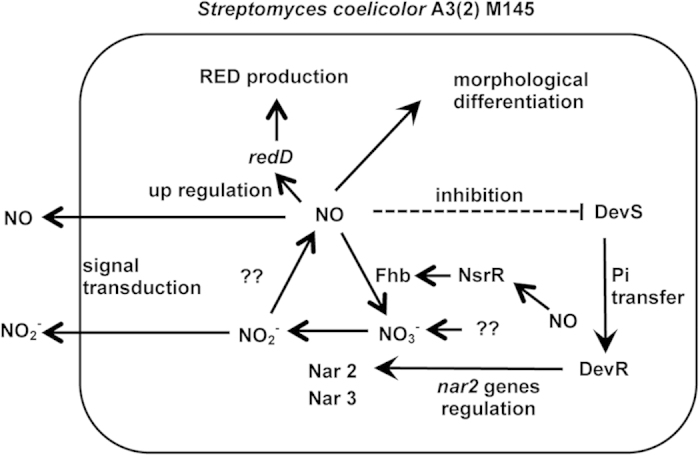
Summary of NO metabolism in *S. coelicolor* A3(2) M145. Nitrate reductase (Nar) and NO dioxygenase flavohemoglobin (Fhb) participated in NO_2_^−^ and NO production, forming a nitrogen oxide cycle (NO_3_^−^ → NO_2_^−^ → NO → NO_3_^−^). NO controls its intracellular concentration by regulating Nar2 (*narG2H2J2I2*) and FhbA gene expressions via transcriptional factors NsrR and DevSR. Endogenously formed NO controlled antibiotic (RED) production and morphological differentiation.

This study proposed a nitrogen oxide cycle that regulates cellular NO levels in *S. coelicolor* and its underlying metabolic and morphogenic mechanisms (Fig. 7). The homeostatic regulation of NO in cells is crucial to understanding the complex life of organisms. To date, endogenous production of NO without any exogenous nitrogen species is known to be achieved by transiently controlled production of NO synthase^19^. The *S. coelicolor* mechanism is unique in that Nar and Fhb play key roles in NO homeostasis. Conventional roles ascribed to Nar and Fhb are dissimilation of NO_3_^−^ and detoxification of NO, respectively, both of which are mechanisms for responding to exogenously added nitrogen oxides in most bacteria. Recent studies showed a similar Nar-dependent NO_2_^−^ production in human pathogen bacterium, *Mycobacterium tuberculosis* (Mtb). The NO_2_^−^ production is believed to be a system for survival of Mtb in host^20^,^21^. We here demonstrated the role of Nar and Fhb in balancing the levels of cellular NO to control cell signaling in the proposed nitrogen oxide cycle mechanism (Fig. 7). The mechanism of *S. coelicolor* does not require exogenous nitrogen oxides, which highly suggests that it is not a mechanism for environmental responses, but is a constitutive housekeeping one.

In this study, we could not identify the mechanism involved in the production of NO_3_^−^ in *S. coelicolor*. Our results indicated that NO_2_^−^ and NO were generated after the production of NO_3_^−^ (Fig. 2), indicating that NO_3_^−^ is the first nitrogen oxide produced among the three nitrogen oxide species (NO_3_^−^, NO_2_^−^, and NO) as the precursor of NO_2_^−^ and NO. To date, few NO_3_^−^ producing enzymes are known except for the enzymes that convert NO and NO_2_^−^ to NO_3_^−^, thereby, indicating that NO_3_^−^ generated in *S. coelicolor* was produced by some unidentified metabolic pathway. Therefore, we propose an extremely interesting topic regarding the production of NO_3_^−^ in *S. coelicolor*.

While NOS and NO-generating nitrite reductase were previously known as the only enzymes to produce NO, recent studies have disclosed several other NO generation mechanisms. Nitric oxide generation from NO_2_^−^ is known to depend on ether enzymatic or nonenzymatic reaction in bacterial cells. It was suggested that *Escherichia coli* and *Salmonella typhimurium* produce NO from NO_2_^−^ by periplasmic cytochrome nitrite reductase^22^ and Nar^23^, respectively. Moreover, other heme-containing^24^,^25^,^26^ proteins (such as hemoglobins and NOS) and molybdenum proteins^27^,^28^ (such as aldehyde oxidase and xanthine oxidase) were shown to convert NO_2_^−^ to NO. Further, nonenzymatic formation of NO from NO_2_^−^ is also known^29^,^30^. Here we demonstrated the NOS-independent NO production in *S. coelicolor* (Fig. 2), its mechanism is to be elucidated.

Only links have been suggested between NsrR or DevSR and tolerance against stress^8^,^31^,^32^,^33^. In *S. coelicolor*, both proteins up- and down-regulate Fhb and Nar gene expressions respectively, to control cellular NO level in response to endogenously produced NO in an auto-regulation mechanism (Fig. 5). Despite NO production from early stages of culture, this mechanism can explain that NO performed as a signaling molecule at the later stage. Disruption of the genes *devS* or *devR* had a significant influence on secondary metabolism (Fig. 4c), indicating high stringency of this regulation system and the importance of endogenous NO. Generally, bacterial cells synchronously start new metabolic processes including secondary metabolism, which is called as quorum sensing^34^. Here, the excreted NO_2_^−^ (or NO derived from NO_2_^−^) influenced FhbA production by neighboring cells (Fig. 6) showing that *S. coelicolor* cells communicate with one another via extracellular NO_2_^−^ or NO derived from NO_2_^−^. Intracellular and extracellular NO_2_^−^ are in equilibrium thus when extracellular NO_2_^−^ concentrations increase, endogenous NO can overcome FhbA, and the cell can start the new metabolic process which can shared between all cells. Thus, NO_2_^−^ acts as an autoinducer in quorum sensing. So it should be NO_2_^−^ that is also the purported signaling molecule.

Furthermore, we found a NO_2_^−^ removal system (Fig. 5c). This NO homeostatic regulation system can explain the gradual decrease of accumulated NO_2_^−^ (Fig. 1a). Thus, the NO_2_^−^ removal system may be important not only to regulate NO homeostasis but also to complete the NO homeostatic regulation system in *S. coelicolor*. However, this notion needs to be proven by additional genetic studies.

The identification of NO as a signaling molecule in *Streptomyces* bacteria and the novel regulation system now allows us to take a step towards a better understanding of the regulation of synthesis of biologically active agents in the producer. In fact, our results show that the production of the antibiotic Red drastically increases depending on the concentration of exogenous NO (Fig. 3). The regulation of NO homeostasis in accordance with various systems continues to be an important subject for further investigation in all organisms to provide a new perspective on NO biology and to contribute towards human welfare.

## Methods

### Bacterial strains, plasmids, and culture conditions

Strains used in this study are listed in Supplementary Table S1. *Streptomyces coelicolor* A3(2) M145 strain (wild-type) was obtained from the John Innes Centre, UK. Mannitol soya flour agar (2% mannitol, 2% soya flour, 2% agar) was used for sporulation, and the strains were routinely grown on YEME-gln (glutamine) solid medium [0.3% yeast extract, 0.5% Bacto-peptone, 0.3% malt extract, 1% glucose, 50 mM L-glutamine (pH 7.2)] at 30 °C. The medium contained no detectable (by ion chromatography or colorimetric analysis) amount of nitrate or nitrite. Minimal solid medium (0.05% L-asparagine, 0.05% K_2_HPO_4_, 0.02% MgSO_4_· 7H_2_O, 0.001% FeSO_4_· 7H_2_O, 1% Glucose, 2% agar) was used for detection of NO_2_^−_^production ability in M145. *E. coli* DH5α (Takara, Kyoto, Japan) was used as the host for routine cloning. Media, culture conditions, and DNA manipulations for *Streptomyces* and *E. coli* were performed as described by Kieser *et al.*^35^ and Green and Sambrook^36^, respectively. Media and culture conditions for the strains used for gene disruption followed a protocol of the REDIRECT PCR-targeting method^37^. *E. coli* HST04 *dam*-/*dcm*- (Takara) was used as a non-methylating cosmid and plasmid donor strain. *E. coli* Origami 2 (DE3) was used for recombinant DevS and DevR production. The plasmids, cosmids (kindly provided by the John Innes Centre, UK), and primers used for this study are listed in Supplementary Tables 2 and 3.

### Construction of mutants and complementation

The open reading frames in the chromosomes were replaced with drug resistance cassettes by using REDIRECT PCR targeting^37^. Each drug resistance cassette flanked by Flippase recognition target (FRT) sites was amplified by PCR using PrimeSTAR GXL DNA polymerase (Takara), and each primer set is listed in Table S2. To obtain a target gene-disrupted version of the mutant cosmids by the λRed system, amplified cassettes were introduced into *E. coli* BW25113/pIJ790^37^ harboring an appropriate cosmid (Supplementary Table 2). The resulting construct was confirmed by PCR and introduced into *E. coli* HST04 *dam*-/*dcm*- (Takara) to obtain a non-methylating cosmid, and each mutated cosmid was introduced into *S. coelicolor* A3(2) M145 or its derivatives by protoplast transformation. Drug‐resistant recombinants (Supplementary Table 1) were screened, and successful recombination was checked by PCR using appropriate primer sets and a complementation study.

To obtain a marker-less mutant, the drug-resistance cassette was eliminated from the corresponding disrupted cosmid by introduction into *E. coli* strain BT340 in which recombination between both FRT mutagenesis cassette-flanking regions was induced by Flippase. In these new cosmids, only 81 base pairs (SCAR) remained in frame with the adjacent ORFs. Each resulting cosmid was introduced into the corresponding mutant and then the drug-sensitive mutant was screened and the replacement of drug-resistant cassette with SCAR was checked by PCR, using the appropriate primer set. Integration plasmids, pTYM19-*narG2H2J2I2*, pKU460-*nsrR-hmpA*, and pKU460-*devS-R*, used for genetic complementation of knockout mutants, were prepared in the following manner. Each gene coding region, *narG2H2J2I2* (SCO0216-0219), *nsrR-hmpA* (SCO7427-7428), and *devS-R* (SCO0203-0204), containing each upstream region (150–300 bp), was amplified by PCR, using the primer sets listed in Supplementary Table 3. The resulting *narG2H2J2I2* fragment was cloned into the *Hin*DIII site of pTYM19^38^. The *nsrR-hmpA* and *devS-R* fragments were cloned into the *Eco*RI/*Hin*DIII site of pKU460^39^, respectively, and the resulting plasmids were introduced into each disruptant.

### Determination of NO_3_^−^ and NO_2_^−^

NO_3_^−^ was determined by ion chromatography, using a 761 Compact IC (Metrohm)^6^. NO_2_^−^ was determined by Griess reagent assay^40^. Both nitrogen oxides were extracted from the medium for determination. Cells were grown for the indicated periods on a cellophane membrane covering the surface of a YEME-gln agar plate. After cultivation, cells on the cellophane were removed, five blocks of 1 × 1 cm were cut out from separate positions on the plate, and the blocks were combined and homogenized in 5 ml distilled water. After centrifugation, the supernatant was further filtered through a 0.45-μm cellulose acetate filter and subjected to determination of nitrogen oxides in the culture medium. The determination depended on a standard curve made using medium containing 0, 1, 10, 25, 50, and 100 μM NO_2_^−^ or NO_3_^−^.

### Enzyme assay

Nar activity was assayed using dithionite/methylviologen as an electron donor, as previously described^6^.

### Detection of FhbA by western blot analysis

Spores of M145 or its derivatives were inoculated on a solid medium plate at intervals of 1 cm (a total of 45 spots) with a toothpick. Culture conditions are presented in figure legends. Cells on the plate were incubated at 30 °C, harvested with a toothpick, and disrupted by sonication in lysis solution (0.3-g urea, 0.15-g thiourea, 0.1-ml 20%CHAPS solution, and 0.001-g DTT, in 1-ml distilled water). Seven micrograms of each soluble protein was separated by 12.5% SDS-PAGE, transferred onto an Immun-Blot PVDF Membrane (Bio-Rad), and probed with rabbit polyclonal FhbA antibody (1:2000). Proteins of interest were detected with goat anti-rabbit (GAR)-HRP conjugate (1: 1000, Bio-Rad) and visualized with the ECL Plus Western Blotting Detection System (GE), according to the provided protocol.

### *In situ* detection of NO

Strains were grown at 30 °C on YEME-gln. Endogenously formed NO was detected using DAF-2DA (Dojindo) as described previously^6^. Photographs were taken with excitation at 495 nm and emission at 515 nm using FLUOVIEW FV300 System (Olympus).

### Isolation of total RNA and qPCR

Total RNA was isolated using RNeasy Kit (Qiagen) from strains grown on cellophane-covered solid medium under several culture conditions, according to the manufacturer’s instructions. Conditions for each culture are given in the figure legends. cDNA was generated using a PrimeScript^®^ RT reagent Kit with gDNA Eraser (Takara) and served as a template for qPCR. The primers used for qRT-PCR are indicated in Supplementary Table 3. qPCR was performed in a Thermal Cycler Dice Real Time System (Takara). PCR mixture (total 25 μl) contained 0.1 μg of generated cDNA, 10 pmol of an appropriate primer set (Table S3), and SYBR^®^ *Premix Ex Taq*^TM^*II* (Takara). The *hrdB* gene of *S. coelicolor* was used as an internal control^41^.

### Determination of RED

Undecylprodigiosin (RED) was determined as described^42^ with some modifications. Spores were inoculated onto a cellophane-covered YEME-gln plate at intervals of 1 cm (total of 45 spots) with a toothpick. After culture at 30 °C for 48 h, cells on cellophane were transferred to a fresh YEME-gln medium plate containing 0, 100, 500, or 1000 μM NO_2_^−^. Before the transfer, 300 μl distilled water was added onto the plate to allow tight contact of cellophane with the agar plate. Cells were incubated at 30 °C for 48 h and then collected and submitted for the determination of RED production. To remove the blue-pigmented antibiotic actinorhodin (ACT) from the cells, 1 M KOH was added and after incubation at 25 °C for 1 h, cells were centrifuged at 8,000 × *g* for 10 min, and the supernatant containing ACT was removed. For RED, the cells were washed twice with 0.9% NaCl after ACT extraction. The resulting pellet was extracted with methanol (pH 2.0, adjusted with HCl) overnight at 25 °C, followed by centrifugation at 8,000 × *g* for 5 min, and absorbance at 530 nm was measured. A molecular extinction coefficient of ε_530_ = 100,500 M^−1^cm^−1^ was used for the determination of RED.

### Overexpression and purification of recombinant proteins

*hmpA (SCO7428)* encoding FhbA*, devR (SCO7428)*, and *devS (SCO7428) genes* were amplified with primeSTAR GXL DNA polymerase, using primers listed in Supplementary Table 3. Each amplified gene was treated with *Nde*I and *Eco*RI and cloned into the corresponding sites of pET28b. Each expression construct was then transformed into *E. coli* Origami 2(DE3). For the overexpression of proteins, 1% of each overnight culture was inoculated into a fresh LB medium (1000 ml) containing 50 μM kanamycin, and after 1 h growth at 37 °C, IPTG [0.2 mM (for FhbA and DevR) or 1 mM (for DevS)] was added to the medium. Each culture was then incubated at 30 °C (FhbA) or 16 °C (DevR and DevS) for 24 h. Grown cells were harvested and resuspended in Tris buffer [20 mM Tris-HCl (pH 8.0); containing 300 mM NaCl, 0.1 mM DTT, 20 mM imidazole, and 10% glycerol] and broken by sonication on ice. After centrifugation at 4 °C, the supernatant was loaded onto a Ni-NTA Agarose column (Qiagen) and washed with the same buffer. Each target protein was eluted with Tris buffer containing 500 mM imidazole. Purified FhbA was used to produce rabbit polyclonal antibody.

### Autophosphorylation of DevS

DevS (1 μg) was incubated with 10 μCi γ^32^P-ATP (Perkin-Elmer) in a 10-μl reaction mixture [containing 50 mM Tris HCl, 50 mM KCl, 5 mM MgCl_2,_ and 10 μM ATP (Sigma, Missouri, USA) (pH 8.0)] at 30 °C for 10, 30, and 60 s, in the presence or absence of NOC5 (NO generator, half-life: 25 min) (Dojindo) with final concentrations of 0.1, 1, and 10 μM, respectively. Then, each mixture was promptly transferred to a heat-block set (56 °C) and 3 μl of stop solution (250 mM Tris-HCl, 10% SDS, 0.5% bromophenol blue, 50% glycerol, 500 mM DTT, pH 6.8) was added. After incubation for 10 min, each reaction mixture was separated by 12.5% SDS-PAGE, and the gel was rinsed twice with water and subjected to autoradiography with BAS-2500 (Fuji film).

### Other analytical methods

Cellular protein was determined using a protein assay reagent (Bio-Rad) after homogenization of cells with an ultra-sonicator as described previously^6^.

## Additional Information

**How to cite this article**: Sasaki, Y. *et al.* Nitrogen oxide cycle regulates nitric oxide levels and bacterial cell signaling. *Sci. Rep.* **6**, 22038; doi: 10.1038/srep22038 (2016).

## Supplementary Material

Supplementary Information
