# Supplementary material for: Nitrogen oxide cycle regulates nitric oxide levels and bacterial cell signaling
Source: Sci Rep. 2016 Feb 25;6:22038. doi: 10.1038/srep22038 (PMC4766573; doi:10.1038/srep22038)
Supplement: Supplementary Information [file srep22038-s1.pdf]

Supplementary Information

Nitrogen oxide cycle regulates nitric oxide levels and bacterial cell signaling

Yasuyuki Sasaki[1*], Haruka Oguchi[1], Takuya Kobayashi[1], Shinichiro Kusama[1], Ryo Sugiura[1], Kenta Moriya[1], Takuya Hirata[1], Yuriya Yukioka[1], Naoki Takaya[2], Shunsuke Yajima[1], Shinsaku Ito[1], Kiyoshi Okada[1], Kanju Osawa[1], Haruo Ikeda[3], Hideaki Takano[4], Kenji Ueda[4], and Hirofumi Shoun[1]

[1]Department of Bioscience, Faculty of Applied Bioscience, Tokyo University of Agriculture, Sakuragaoka Setagaya-ku, Tokyo 156-8502, Japan, [2]Faculty of Life and Environmental Sciences, University of Tsukuba, Tsukuba, Ibaraki, Japan, [3]Laboratory of Microbial Engineering, Kitasato Institute for Life Sciences, Kitasato University, 1-15-1 Kitasato, Sagamihara, Kanagawa 228-855, Japan, and [4]Life Science Research Center, College of Bioresource Sciences, Nihon University, 1866 Kameino, Fujisawa 252-0880, Japan

*To whom correspondence should be addressed:
Yasuyuki Sasaki, phD
Department of Bioscience, Faculty of Applied Bioscience, Tokyo University of Agriculture, Sakuragaoka Setagaya-ku, Tokyo 156-8502, Japan
Tel/Fax: +81-3-5477-2365; E-mail: y1sasaki@nodai.ac.jp

24 (h)

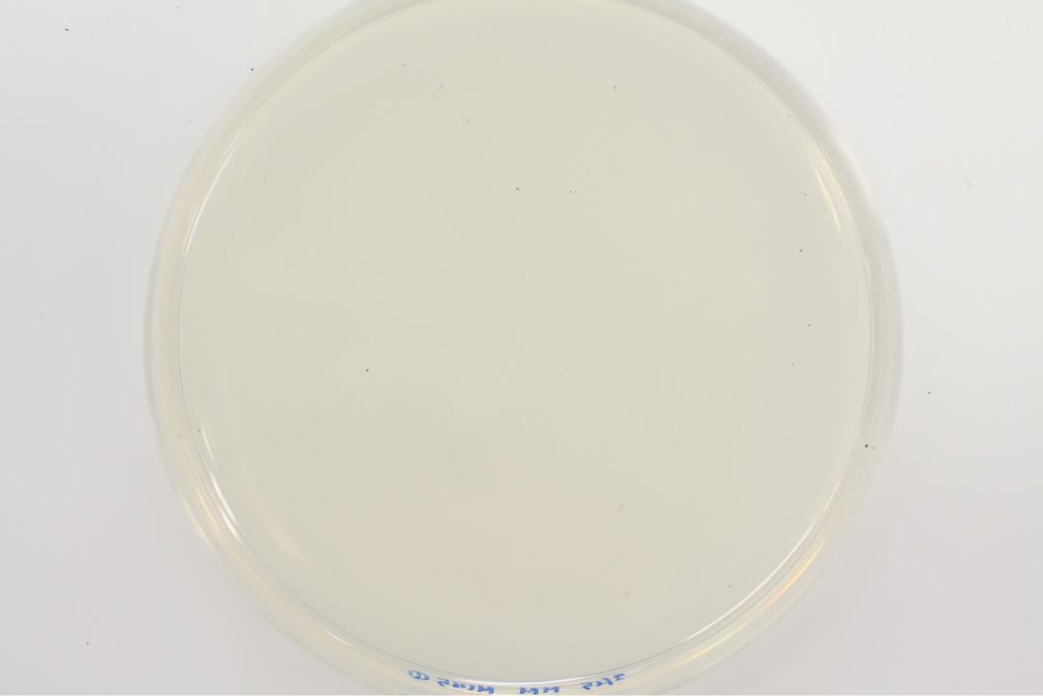

48 (h)

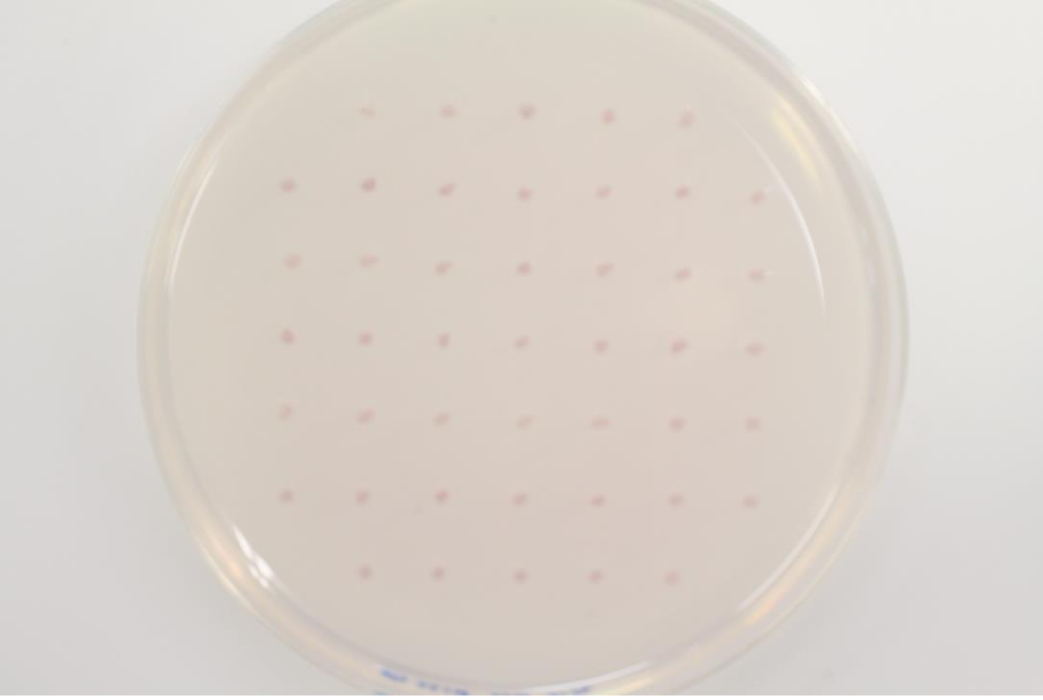

Supplementary Figure 1. *S. coelicolor* converts organic nitrogen to nitrite. M145 cells were grown on chemically defined medium for the indicated period at 30°C and NO_2^- was stained as in Figure 1c.

```
mDevS      MTTGGLVDENDGAAMRPLRHTLSQLRLHELLVEVQDRVEQIVEGRDRLDGLVEAMLVVTA
sDevS      --------MENAEETREARVRLPQLRLDELLEELQARLDAARGTRDRVHSLLEAVLSVGR
                    ::.   *  *  *.****.*** *:* *::     ***:..*:**:* *
mDevS      GLDLEATLRAIVHSATSLVDARYGAMEVHDR-QHRVLHFVYEGIDEETVRRIGHLPKGLG
sDevS      ELDLEQALRSIVEAAAALVDAEYAALGVIGPDGKRLSAFHTVGVDAQQIARIGPYPEGHG
            **** :**:**.:*::****.*.*: * .   :*:  *   *:* : : ***  *:* *
mDevS      VIGLLIEDPKPLRLDDVSAHPASIGFPPYHPPMRTFLGVPVRVRDESFGTLYLTDKTNGQ
sDevS      ILGELIRHPEPLRLAKLSDHPASYGFPAHHPPMNTFLGVPIRVRDHVFGNLYLTEKRGGQ
           ::* **..*:**** .:* **** ***.:****.******:****. **.****:* .**
mDevS      PFSDDDEVLVQALAAAAGIAVANARLYQQAKARQSWIEATRDIATELLSGTEPATVFRLV
sDevS      QFDEDDESVLATLAVAAGVAIDNARLYEESRLRERWLQVNAEITHTLMSGADQGGVLPLI
            *.:*** :: :**.***:*: *****:::: *: *::.. :*:  *:**:: . *: *:
mDevS      AAEALKLTAADAALVAVPVDEDMPAADVGELLVIETVGSAVASIVGRTIPVAGAVLREVF
sDevS      AERAREITGSALSVVATPVSG------TDTLAVELAVGHEADAWRGIVLPVEGTLIGQAF
           * .* ::*.:  ::**.**.       .. * *  :**  . :  * .:** *::: :.*
mDevS      VNGIPRR-----VDRVDLEGLDELADAGPALLLPLRARGTVAGVVVVLSQGGPGAFTDEQ
sDevS      VQRAPVHSADVCRDSRVSAGPPRFEGLGPGVAVPIGSSAEARGVVLLVRQSGGQEFSEEE
           *:  * :      *     *  .: . **.: :*: : . . ***::: *.*   *::*:
mDevS      LEMMAAFADQAALAWQLATSQRRMRELDVLTDRDRIARDLHDHVIQRLFAIGLALQGAVP
sDevS      TEPLLVFAAQAAVAMELAERRADAEQIALLEDRDRIARDLHDLAIQRLFATGMTLQSAGR
            * : .** ***:* :**  :   .:: :* *********** .****** *::**.*
mDevS      HERNPEVQQRLSDVVDDLQDVIQEIRTTIYDLHG-ASQGITRLRQRIDAAVAQFADS-GL
sDevS      RVQDPMASERILRAVDDLDETIKIIRSTIFGLRSRDDDAVPGLRSRAVRAVGEAAPLLGF
           : ::* ..:*:  .****::.*: **:**:.*:.  .:.:. **.*   **.: *   *:
mDevS      RTSVQFVGPLS-VVDSALADQAEAVVREAVSNAVRHAKASTLTVRVKVD-DDLCIEVTDN
sDevS      APSVRMEGLLDTHVPARTADHVMAVLTESLTNVARHARADRADVVLETDGKQVRLTVTDN
            .**:: * *.  * :  **:. **: *:::*..***:*.   * ::.* .:: : ****
mDevS      GRGLPDEFTGSGLTNLRQRAEQAGGEFTLASVPGASGTVLRWSAPLSQ------------
sDevS      GVGIPDGGRRSGLTNMAERAQKLGGGMDLES-PGGKGTRLVWHAPLADSPDRAEGGRSAR
           * *:**    *****: :**:: ** : * * **..** * * ***::


mDevR      ------------MVKVFLVDDHEVVRRGLVDLLGADPELDVVGEAGSVAEAMARVPAARP
sDevR      MAHSEQGDNTGSPVRVFLLDDHEVVRRGVHDLLDDEPDITVVGEAATVEQALVRVPALRP
                        *:***:*********: ***. :*:: *****.:* :*:.**** **
mDevR      DVAVLDVRLPDGNGIELCRDLLSRMPDLRCLILTSYTSDEAMLDAILAGASGYVVKDIKG
sDevR      DVAVLDVRLPDGDGVTVCRELRSQMPDLACLMLTSFDDEEALLDSIMAGAAGYVLKQIQG
           ************:*: :**:* *:**** **:***: .:**:**:*:***:***:*:*:*
mDevR      MELARAVKDVGAGRSLLDNRAAAALMAKLRGAAEK--QDP--LSGLTDQERTLLGLLSEG
sDevR      SDLVSAVRTVARGQSLLDASATTKLMARLRGGGQQPVEEPEMLPGLTDREREILELIGEG
            :*. **: *. *:****  *:: ***:***..::  ::*  *.****:** :* *:.**
mDevR      LTNKQIADRMFLAEKTVKNYVSRLLAKLGMERRTQAAVFATELK-RSRPPGDGP
sDevR      LTNRQIGQRLYLAEKTVKNHISRLLAKLGVERRIQAAVIATQARDRMRTEGQN-
           ***:**.:*::********::********:*** ****:**: : * *. *:.
```

Supplementary Figure 2. Protein sequence alignments of DevS and DevR homologs. Shown are amino acid sequences of *S. coelicolor* (sDevS, sDevR) and *Mycobacterium tuberculosis* (mDevS, mDevR). The comparison was done using the ClustalW multiple sequence alignment program.

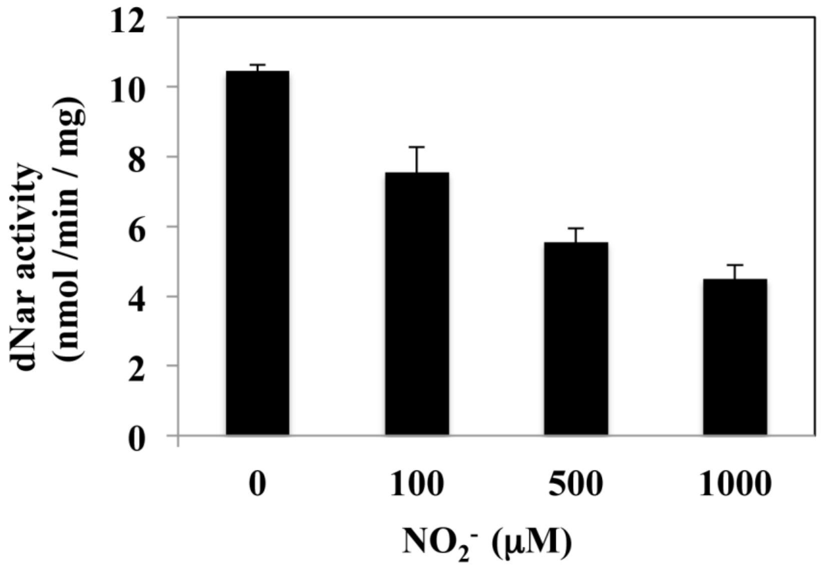

Supplementary Figure 3. Exogenous NO_2^- reduces Nar activity *in vivo*. Cells were grown on plates containing several concentrations of NO_2^- (0, 100, 500, and 1,000 µM, respectively) as in Fig. 1. Membrane fractions were prepared from the cells and used to determine dNar activity. Error bars indicate standard deviation (n=3).

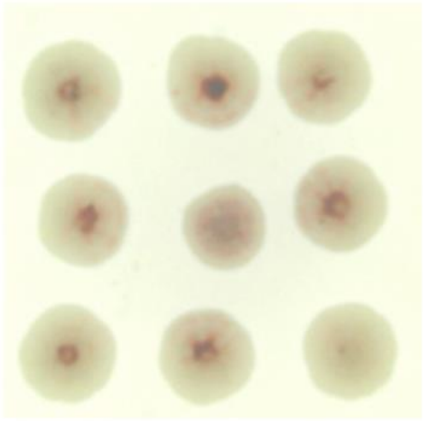

Supplementary Figure 4. Red-producing ability of the *ΔnarG/G2/G3* mutant strain was not restored by excreated NO_2^- from M145. M145 was inoculated in all spots except the center spot in which the *ΔnarG/G2/G3* mutant was inoculated onto the plate (a total of 9 spots) and incubated for 72 h.

Supplementary Table 1. Strains used in this study.

Strain	genotype, characteristic	Reference
Streptomyces coelicolor A3(2)		
M145 (wild type)	SCP1¯SCP2¯	11
ΔnarG	M145 SCO6535::*aac(3)IV* (removing *narG*)	This study
ΔnarG2	M145 SCO0216::*aac(3)IV* (removing *narG2*)	This study
ΔnarG3	M145 SCO4947::*aac(3)IV* (removing *narG3*)	This study
ΔnarG2/G3	M145 SCO0216::*aac(3)IV* SCO4947::scar (removing *narG2G3*)	This study
ΔnarG/G2/G3	M145 SCO6535::*hyg*/SCO0216::scar/SCO4947::scar (removing *narGG2G3*)	This study
ΔnarG/G2/G3:: nar2	M145 SCO6535::*hyg*/SCO0216::scar/SCO4947::scar/pTM19-*nar2operon*	This study
ΔhmpA	M145 SCO7428::*aac(3)IV* (removing *narG*)	This study
ΔhmpA:: hmpA	M145 SCO7428::*aac(3)IV* /pKU460-SCO7427-7428 (complementation of *hmpA*)	This study
ΔdevS	M145 SCO0203::*aac(3)IV* (removing *devS*)	This study
ΔdevS::devR-S	M145 SCO0203::*aac(3)IV* /pKU460-SCO0204-0203 (complementation of *devS*)	This study
ΔdevR	M145 SCO0204::*aac(3)IV* (removing *devR*)	This study
ΔdevR::devR-S	M145 SCO0204::*aac(3)IV* /pKU460-SCO0204-0203 (complementation of *devR*)	This study
ΔredD	M145 SCO5877::*aac(3)IV* (removing *redD*)	This study

Supplementary Table 2. Primers used for gene disruption.

Primer Name	Cosmid[a]	Sequence (5′-3′)[b]	Position (nt)[c]
ΔnarG F	St5C7	GCGAGCACCCGGGACCGGGTGAGCAAGGGAGCGCACATG attccggggatccgtcgacc	7230284–7230252
ΔnarG R	St5C7	GGTTCATGACCATCGCAACCTGGGCCATCGGGCGCATCA tgtaggctggagctgcttc	7226517–7226555
ΔnarG2 F	StJ12	GGTCCGGCGCGACCCCCGCGCCTAGCGTTCTGGCCCATG attccggggatccgtcgacc	206444–206482
ΔnarG2 R	StJ12	ATGACGCGTCCGACGGTGGCTTCGTCTCGGGACATGTCA tgtaggctggagctgcttc	210217–210179
ΔnarG3 F	StK13	GGAGATGGCAGCGTGGTGCGAAGCAGGGCGAAGGCCATG attccggggatccgtcgacc	5381112–5381150
ΔnarG3 R	StK13	GGTTCATGACCATCGCCACCTGTGCCATGACGCGCATCA tgtaggctggagctgcttc	5384867–5384829
ΔhmpA F	St5C11	GGTTCATGACCATCGCCACCTGTGCCATGACGCGCATCA tgtaggctggagctgcttc	8242319–8242357
ΔhmpA R	St5C11	GAGTTTTGCGGCGGGCGCCGTCGGCCGCCACCGGCGTCA tgtaggctggagctgcttc	8243587–8243555
ΔnsrR F	St5C11	GGCGAACCTAGCATGCGCATTTGATAGCGTCCTGGTATG attccggggatccgtcgacc	8241799–8241834
ΔnsrR R	St5C11	GGCCACAGCTCGACGGCCTCAGGGGGCCGCCGCCCGTCA tgtaggctggagctgcttc	8242317–8242279
ΔdevS F	StJ12	GCTGCCGGCAAGCGGTGAGGCGTTGGAGGAGCAGCGATG attccggggatccgtcgacc	194673–194638
ΔdevS R	StJ12	AGTGATCGGGTGCCGTCCGTCCACCGACGCCGTGCGTCA tgtaggctggagctgcttc	192844–192882
ΔdevR F	StJ12	TTCCGACACGGGCCGAGACATCGCGAGGAGCATCCGATG attccggggatccgtcgacc	195497–195459
ΔdevR R	StJ12	ATCGGCGCCGTCACGCGACCCGACCGGCCGGAATCGTCA tgtaggctggagctgcttc	194724–194762
ΔredD F	St2E9	GTCCTGTGTTGAGGCCCGAATCCGATCGTTCGGTGGATG attccggggatccgtcgacc	6432530−6432568
ΔredD R	St2E9	TTCACGTGGGCACCGCCCGCGGCCGGCCCCGGGTCGTCA tgtaggctggagctgcttc	6433654-6433616

[a]St were provided by JIC. [b]Sequences corresponding to the drug-resistance gene are shown in lower case. [c]Corresponding position in the geneme sequence databese of *S. coelicolor* (http://www.ncbi.nlm.nih.gov/).

Supplementary Table 3. Primers used for genetic complementation, transcriptional analysis, production of recombinant proteins, and gel-shift assay.

Primer Name	Sequence (5′-3′)[a]	Position (nt)[b]	Used for
comp nar2 F	CTCGAGAAGCTTAGTGTGCGGGGCCGCGCCCGT	206180–206200	complementation
comp nar2 R	CTCGAGAAGCTTTCATACGGGGTGCCGGTCGCGCC	213321–213299	complementation
comp hmpA F	GAATTCTGGCGTCGAGCTGCCGGCC	8241535–8241553	complementation
comp hmpA R	AAGCTTCTACTGCTGTCCGAGCCAGAGG	8243551–8243530	complementation
comp devR/S F	GAATTCCGAGGACGCCGTCGACG	195761–195745	complementation
comp devR/S R	AAGCTTCGGACCGGCCGCCTTC	192888–192903	complementation
narG RT F	CACCCGACCTGCACAGCGTCGGA	7230166–7230144	RT-PCR
narG RT R	CACGCGAGCACCGGGTCCGTC	7229812–7229832	RT-PCR
narG2 RT F	CGCCCTGCCTCGTTTCGACGAAGGT	207796–207820	RT-PCR
narG2 RT R	AGGGCGATCATCGAACGGCCACCC	208098–208075	RT-PCR
narG3 RT F	ACACGGGCGGCCGCGGTGACAAC	5382424–5382446	RT-PCR
narG3 RT R	ATGACGATCATGCAGCGGCCCCGGGTCT	5382748–5382721	RT-PCR
hrdB qPCR F	GCATGCTCTTCCTGGACCTCAT	6368554–6368575	qPCR
hrdB qPCR R	TGGAGAACTTGTAGCCCTTGGTGTA	6368646–6368622	qPCR
narG3 qPCR F	AGACCCAGCAGACGGATT	5381371–5381388	qPCR
narG3 qPCR R	GAATAGGTGTACCAGGAAAAGGAG	5381470–5381447	qPCR
narG2 qPCR F	GTTCACCTACGCCTTCAACTACTA	210088–210111	qPCR
narG2 qPCR R	CAGTACTCCACTCGCTGGTC	210180–210161	qPCR
rFhb F	CATATGCTCTCCGAACAGT	8242355–8242370	recombinant
rFhb R	GAATTCGCTCGGACAGCAGTAG	8243536–8243551	recombinant
rDevR F	CATATGGCGCACAGCGAGCAGGGC	195458–195441	recombinant
rDevR R	GAATTCTCAGTTCTGGCCTTCGGTGC	194760–194779	recombinant
rDevS F	CATATGGAGCAGATCGCCCTGCTGGAGGAT	193527–193504	recombinant
rDevS R	GAATTCACGGGCGGACCGGCCGCCTT	192883–192902	recombinant
-500 nar2 F	GCCGAAGGGGCCCAGGAG	205994–206011	gel shift assay
-500 nar2 R	CCTCACGGCGCGTCAGCA	206570–206553	gel shift assay

[a]Restriction site are underlined. [b]Corresponding position in the genome sequence database of *S. coelicolor* (http://www.ncbi.nlm.nih.gov/).
